# Effects of a Prolonged-Release Protein Substitute on 24-h Phenylalanine and Tyrosine Profiles in Phenylketonuria: A Randomized Crossover Study

**DOI:** 10.3390/nu18162738

**Published:** 2026-08-21

**Authors:** Valentina Rovelli, Graziella Cefalo, Alice Re Dionigi, Annalisa Finizii, Sabrina Paci, Juri Zuvadelli, Giuseppe Banderali

**Affiliations:** Clinical Department of Pediatrics, University of Milan, ASST Santi Paolo e Carlo, San Paolo Hospital, 20100 Milan, Italy; graziella.cefalo@asst-santipaolocarlo.it (G.C.); alice.redionigi@asst-santipaolocarlo.it (A.R.D.); finizii.annalisa@gmail.com (A.F.); sabrina.paci@asst-santipaolocarlo.it (S.P.); juri.zuvadelli@asst-santipaolocarlo.it (J.Z.); giuseppe.banderali@unimi.it (G.B.)

**Keywords:** phenylketonuria (PKU), blood phenylalanine, phenylalanine/tyrosine ratio, prolonged-release amino acids, prolonged-release protein substitute, metabolic control

## Abstract

Background/Objectives: Achieving optimal metabolic control in phenylketonuria (PKU) remains challenging, particularly in adolescents and adults. Conventional free amino acid–based protein substitutes are absorbed rapidly, whereas prolonged-release formulations are designed to provide a more gradual amino acid release profile. Whether this translates into more favorable 24 h Phe and Tyr profiles and improved short-term biochemical parameters of metabolic control remains uncertain. This study evaluated whether a prolonged-release amino acid–based protein substitute (PR-AA) could improve 24 h metabolic control compared with standard protein substitutes. Methods: This randomized, controlled, open-label crossover study included adolescents and adults with PKU aged ≥16 years. Participants received either conventional protein substitutes (standard of care; SC) or PR-AA three times daily for two consecutive days, followed by crossover to the alternate treatment. Blood spot samples were collected at five time points over 24 h on the second day of each treatment period. The primary outcome was the 24 h blood Phe profile. Secondary outcomes included blood Tyr, Phe/Tyr ratio, and exploratory assessment of branched-chain amino acids (BCAAs). Results: Twenty-one patients were enrolled, of whom 16 were included in the intention-to-treat analysis and 12 in the per-protocol analysis. PR-AA treatment resulted in significantly lower mean blood Phe levels over 24 h compared with SC (522 ± 194 vs. 561 ± 165 µmol/L; p = 0.0009), corresponding to a reduction of approximately 6–7%. The effect was consistently observed throughout the 24-h assessment period, particularly during the early morning, and became detectable after only two days of treatment. PR-AA was also associated with significantly higher blood Tyr levels (approximately +18–20%; p < 0.05) and a lower Phe/Tyr ratio (approximately −20%; p < 0.001) compared with SC. Exploratory analyses demonstrated modestly lower and more stable BCAA profiles with PR-AA. No significant carryover effects or treatment-related safety concerns were observed. Conclusions: Short-term treatment with a prolonged-release protein substitute was associated with modest but consistent improvements in short-term biochemical parameters of metabolic control in adolescents and adults with PKU, including lower blood Phe and higher Tyr levels, resulting in a reduced Phe/Tyr ratio. These findings suggest that optimizing amino acid delivery kinetics may represent a promising strategy to improve short-term metabolic control in PKU. Given the preliminary nature of these findings and the limited number of participants who completed the study protocol, larger studies with longer follow-up, including direct clinical and neurocognitive outcomes and formal variability metrics, are needed to determine the clinical relevance of these biochemical effects.

## 1. Introduction

Phenylketonuria (PKU) is a rare autosomal recessive inherited metabolic disorder caused by mutations in the gene encoding phenylalanine (Phe) hydroxylase (PAH), the enzyme responsible for converting Phe to tyrosine (Tyr) [1,2]. Impaired PAH activity leads to the accumulation of Phe and reduced Tyr availability [3], resulting in neurotoxic effects, particularly in the developing brain, and contributing to neurocognitive dysfunction [4].

Early detection through newborn screening and lifelong treatment have significantly improved outcomes [1,2,5]. Current management primarily relies on a Phe-restricted diet supplemented with Phe-free or low-Phe amino acid mixtures, which remains the cornerstone of therapy for most patients [6]. Although alternative treatments such as large neutral amino acids (LNAA) and pharmacological therapies (e.g., sapropterin, pegvaliase, and sepiapterin) are available, their universal use is limited by factors including variable response, age restrictions, and access [7,8,9,10,11,12,13,14]. Therefore, maintaining metabolic control through dietary management remains essential across the lifespan.

Despite treatment, achieving optimal metabolic control remains challenging, particularly in adolescence and adulthood, where adherence declines and blood Phe concentrations frequently exceed recommended ranges [15,16,17,18,19,20,21,22,23,24]. This age-related deterioration in metabolic control has been linked to a range of neurocognitive, psychological, behavioural, and emotional impairments, particularly among adolescents and adults with PKU, despite continuous treatment [4,24,25,26,27,28,29,30,31,32,33,34,35,36,37,38,39].

Although current therapeutic strategies primarily aim to maintain blood Phe levels within the recommended therapeutic range [5], the concepts of blood Phe stability and fluctuations have increasingly been discussed in the PKU literature as potentially relevant aspects of metabolic control. Emerging evidence suggests that blood Phe stability, fluctuations, and the Phe/Tyr ratio may provide complementary information on metabolic control and have been associated with long-term cognitive, neurological, and psychological outcomes in patients with early-treated PKU [26,40]. Increased fluctuations in blood Phe have been associated with poorer cognitive performance, executive dysfunction, and psychological symptoms in both pediatric and adult populations [40,41,42,43,44,45]. In addition to Phe stability, elevated Phe/Tyr ratio has also been associated with adverse neurocognitive and psychological outcomes and is increasingly regarded as a critical biomarker of metabolic control [46,47,48].

These findings highlight the need for therapeutic approaches that not only lower Phe concentrations but also improve the overall biochemical profile of metabolic control. In this context, the kinetics of amino acid absorption may play a critical role. Free amino acids, as provided in conventional protein substitutes, are rapidly absorbed, leading to faster and higher peaks in plasma amino acid concentrations followed by rapid declines [49,50,51,52]. This pattern is associated with increased amino acid oxidation and less efficient utilization compared with the gradual and sustained absorption observed with intact proteins [53,54,55,56].

Based on this rationale, strategies aimed at modulating amino acid absorption kinetics may help provide a more sustained systemic amino acid availability and, consequently, reduce fluctuations in protein turnover and endogenous Phe release. Prolonged-release amino acid formulations, which involve coating free amino acids with fibre-based excipients such as ethylcellulose and sodium alginate, have been developed to address this limitation by enabling a more gradual gastrointestinal release, thereby providing a more gradual gastrointestinal release of amino acids than conventional free amino acid mixtures [57,58,59]. Preclinical and clinical studies in animal models [57] and healthy individuals [60] have shown that such formulations result in lower peak plasma amino acid concentrations, more sustained absorption, and improved nitrogen utilization, without altering overall exposure. These effects suggest a more physiologic metabolic profile compared with conventional amino acid mixtures. However, their potential impact on 24 h Phe and Tyr profiles and short-term biochemical parameters of metabolic control in individuals with PKU remains to be fully elucidated.

Given that protein metabolism in individuals with PKU shares several core characteristics with that of healthy individuals [61,62], it is reasonable to hypothesize that the metabolic advantages observed with prolonged-release amino acids in healthy populations may also apply to the PKU population. Accordingly, the primary objective of this study was to evaluate the effects of a prolonged-release protein substitute on 24-h blood Phe and Tyr profiles compared with conventional protein substitutes (standard of care), as indicators of short-term biochemical parameters of metabolic control. Secondary objectives included the assessment of additional metabolic parameters, including branched-chain amino acids, as exploratory markers of protein metabolism.

## 2. Materials and Methods

### 2.1. Study Products

The study product was a Phe-free prolonged-release protein substitute (PR-AA; APR Applied Pharma Research, Balerna, Switzerland) available in two formulations: granules and chewable tablets. It consists of amino acids coated with a fiber-based ethyl cellulose and sodium alginate layer, which masks the taste and odor of the free amino acids. This coating technology also enables a more gradual release of amino acids from the formulation (up to 7 h), more closely mimicking the physiological absorption of amino acids from natural dietary protein sources [60].

The study products are intended for use in patients with PKU aged ≥16 years. The granule formulation provided 20 g protein equivalent per 31.5 g sachet, supplemented with vitamins and minerals, whereas the chewable tablets contained amino acids only, corresponding to 1 g protein equivalent per tablet (1.65 g). The nutritional composition of both products is presented in Supplementary Table S1.

For comparison, the standard of care (SC) was either a Phe-free L-amino-acid–based (AA) (*n* = 14) or a low-Phe casein glycomacropeptide (cGMP) based protein substitute (*n* = 7). For participants receiving cGMP-based protein substitutes as standard-of-care, the residual Phe content of the product was accounted for within the individualized prescribed daily Phe allowance (Supplementary Table S2). Total daily Phe intake and total protein equivalent intake were maintained stable across both treatment periods.

### 2.2. Participants

Patients with a confirmed diagnosis of PKU were eligible for inclusion if they were aged ≥16 years, had a mean blood Phe value > 360 µmol/L over the previous 12 months (calculated from at least three samples, with the most recent sample preferably obtained within 30 days prior to study enrollment), and were taking a Phe-free amino acid-based and/or cGMP-based protein substitute. Disease severity was classified according to the BIOPKU database classification based on diagnostic/neonatal blood Phe concentrations. Although some participants were classified as mild HPA according to this system, all participants required dietary treatment and protein substitute supplementation at study enrolment because they were unable to maintain blood Phe levels within the recommended therapeutic target range on an unrestricted diet.

Exclusion criteria included known hypersensitivity to any excipients or components of the investigational product, treatment with any drug therapy for PKU (e.g., sapropterin, Pegvaliase), using the study product as their usual protein substitute, taking LNAA supplementation, pregnancy (confirmed with a pregnancy test) or breastfeeding during the study period, the presence of a comorbidity that may interfere with the study procedures or study outcome, inability to understand and comply with the study requirements, and participation in any other clinical studies concomitantly or within the last 3 months prior to study entry.

### 2.3. Study Design

**Figure 1:**
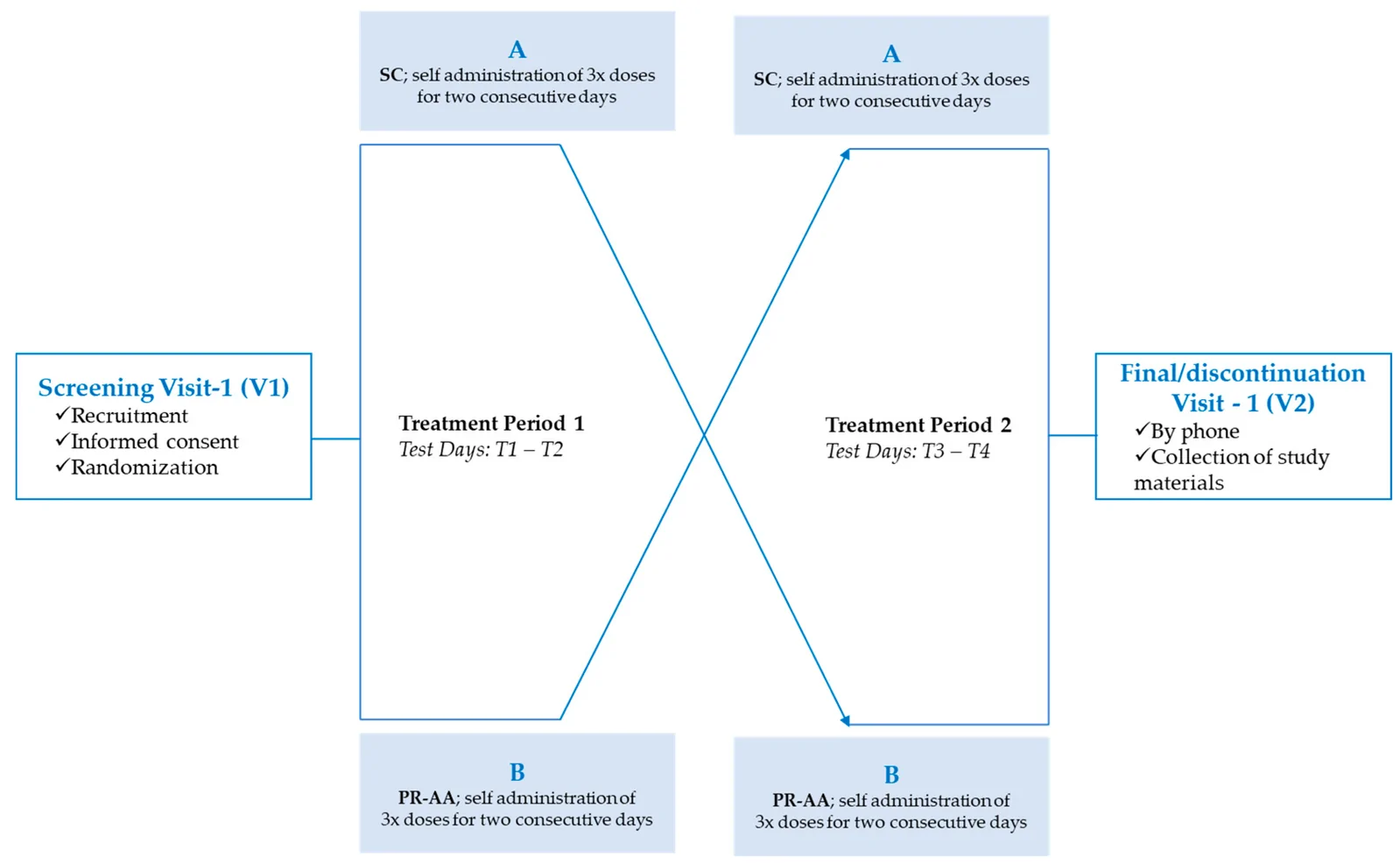
Study design. Abbreviations: PR-AA, prolonged-release amino acid-based protein substitute; SC, standard of care (usual protein substitute).

This was a randomized, controlled, crossover study to primarily evaluate the effects of a PR-AA protein substitute on short-term biochemical parameters of metabolic control, including 24 h blood Phe and Tyr profiles, compared to standard of care (SC) in patients with PKU aged ≥16 years. Patients were randomized to one of two treatment sequences: AB or BA, where A represented the standard of care (AA/cGMP protein substitute) and B represented PR-AA protein substitute (Figure 1).

*Sequence AB:* Patients received 3× daily self-administrations of their usual protein substitute (SC) for two consecutive days during the 1st treatment period, followed by 3× daily self-administrations of PR-AA protein substitute for two consecutive days during the 2nd treatment period.

*Sequence BA:* Patients received 3× daily self-administrations of PR-AA protein substitute for two consecutive days during the 1st treatment period, followed by 3× daily self-administrations of their usual protein substitute (SC) for two consecutive days during the 2nd treatment period.

The study design included a screening/randomization visit (V1), two treatment periods each comprising two test days (period 1: T1–T2 and period 2: T3–T4), and a final/discontinuation visit (V2) conducted at the end of the second treatment period. Test days (T1–T2 and T3–T4) were scheduled on two consecutive days, preferably non-working or non-school days, and ideally over two consecutive weeks. Treatment days in both periods were required to fall on the same days of the week. Test Day 1 (T1) occurred within two weeks of V1.

### 2.4. Randomization and Blinding

The patients were randomized in a 1:1 ratio to one of two treatment sequences (AB or BA). Randomization was performed via a web-based interactive randomization system embedded in the electronic case report form, based on a computer-generated random sequence with a random block size. Participants were allocated following confirmation of eligibility and provision of informed consent. Given the open-label design of the trial, blinding of participants, investigators, and outcome assessors was not possible. Trial registration: This study was registered at ClinicalTrials.gov (ID: NCT05827536; registered on 24 March 2023, with results posted on 30 December 2025).

### 2.5. Interventions

The main prescribed formulation of the study product (PR-AA) was granules. The dose of the study product was adjusted according to body weight (g/kg/day) and matched the protein equivalent provided by each participant’s usual protein substitute intake.

The total daily dose of both the study product (PR-AA) and the usual protein substitute (SC) was divided into three equal portions. The first dosage was taken in the morning (08:00) after an overnight fast of 10–12 h and prior to any food intake, while the second and third dosages were administered at lunch (at 12:00) and dinner (at 20:00).

All dosages were self-administered at home by the patients on both test days of each treatment period (period 1: T1–T2 and period 2: T3–T4), according to the assigned randomization sequence. At each administration, the patient was required to weigh and consume the prescribed amount in accordance with the instructions provided in the study protocol. Meals were provided only within specified time windows and coincided with designated administration times, with no food allowed outside these periods.

Modifications to the randomization sequence, total daily dose, or to the amount of protein substitute (PR-AA or SC) administered at each dosing time, as well as delays in dosing (e.g., postponement of test days) were not permitted.

### 2.6. Study Outcomes

The primary efficacy parameter was blood Phe level, measured at five time points over a 24 h period: at 08:00, 12:00, 16:00, 20:00 on the second test day of each treatment period (T2 and T4), and at 08:00 the following day. Secondary outcome measures were: blood Tyr levels measured at the same five time points; blood Phe/Tyr ratio calculated by dividing the Phe values at various time points by the corresponding Tyr values; and safety parameters, including occurrence of adverse events, anthropometric measurements, medical history and physical examination, treatment compliance, dietary adherence, and concomitant medications.

Exploratory efficacy parameters included blood levels of BCAAs (leucine, isoleucine, and valine), measured at the same five time points over 24 h, to assess additional metabolic responses to the study product. Analyses of exploratory parameters were descriptive in nature and intended to inform the design of future confirmatory studies.

### 2.7. Data Collection

Patient recruitment was initiated on 30 October 2023 and completed on 27 November 2024. At the initial screening visit (V1), written informed consent was obtained and eligibility was confirmed. Data collected at baseline included demographics (age and sex); disease and medical history (date of diagnosis, initiation of dietary treatment, current standard of care, and prior or concomitant treatments for PKU within the previous three months); anthropometric measures (weight, height and body mass index); and dietary patterns and preferences, including food, calorie and nutrient intakes.

Participants were informed about the study and trained in all study-related procedures, including preparation and self-administration of protein substitutes, blood spot collection and storage, and completion of patient diaries, to ensure standardized data collection and adherence to the study protocol. In addition, each patient received nutritional counselling to standardize dietary intake on test days, and dietary instructions were provided.

The final study visit (V2) was conducted remotely (by phone) at the end of the second treatment period, within two weeks after the last test day. The investigator arranged for the collection of blood spot samples from the patient’s home via a dedicated courier service. If paper diaries were used, these were also retrieved.

Patients who discontinued the study prematurely were asked to participate in a remote discontinuation visit, ideally within two weeks of their last test day.

#### 2.7.1. Blood Collection Procedures

A total of five blood spot samples were collected over a 24 h period, prior to product self-administration and before any food intake, on the second test days (T2 and T4) at 08:00, 12:00, 16:00, and 20:00, and at 8:00 on the following morning (±15 min for all time points).

All blood spot samples were collected prior to protein substitute (PR-AA or SC) and food intake. Sample collection was performed by the patients using filter paper (Guthrie cards) provided at the screening visit, in accordance with instructions given by the investigator. A drop of capillary blood obtained via fingertip prick was applied to the Guthrie card. The cards were then allowed to dry at room temperature for approximately three hours, protected from heat sources and direct light. Once dried, the cards were stored at room temperature until shipment to the center laboratory for analysis.

On the days preceding each test day, patients followed their usual standard diet and consumed their usual protein substitute. On test days (T1, T2, T3, and T4), each patient followed a standardized diet with consistent food composition, caloric intake, and nutrient distribution. Diets were tailored according to the patient’s age and body weight. The assigned protein substitute (either PR-AA or SC) was the only protein substitute permitted during the test days. Sport activities were not allowed on sample collection days.

#### 2.7.2. Patient Diaries

Electronic or paper diaries were used to record adverse events, concomitant medications, food intake, daily activities and dietary compliance on each test day (T1, T2, T3 and T4). Dietary compliance was assessed using patient records by comparing the amount of protein substitute weighed with the amount ingested. Information on the timing of blood spot collection and the occurrence of any missing blood spots was also collected.

#### 2.7.3. Safety Assessment

A safety assessment was performed including adverse event monitoring and physical examination. Adverse events were recorded until 30 days after the final dose of the study product. Beyond this period, only serious adverse events suspected to be related to the study product were required to be reported.

### 2.8. Statistical Analysis

#### 2.8.1. Sample Size Calculation

The sample size was calculated based on the primary study outcome, considering data published by Daly et al. [63] and previous findings related to the study product. Given the randomized, controlled, crossover design comparing the efficacy of PR-AA with SC, the study was powered to detect a statistically significant effect of PR-AA on 24 h blood Phe and Tyr profiles compared with standard of care.

A total sample size of 20 patients was determined to be sufficient to detect a between-treatment difference of 30 µmol/L in blood Phe levels, assuming a standard deviation of 40 µmol/L at any blood spot time point. This calculation was based on 80% statistical power and a two-sided significance level of 5%. Sample size calculation was performed using the Proc Power procedure in SAS for a crossover design (paired data).

#### 2.8.2. Data Analysis

Descriptive statistics were calculated for all variables. Quantitative variables are presented as mean ± standard deviation (SD), median, range and 95% confidence intervals (CI). Qualitative variables are summarized as counts and percentages with 95% CI. Percentages include patients with missing data unless otherwise specified.

All analyses were conducted according to the intention-to-treat (ITT) principle. The ITT population included all patients who received at least one dose of the study treatment, irrespective of protocol deviations. The per-protocol (PP) population comprised patients with adequate treatment compliance and complete primary efficacy data, without major protocol deviations, and was used to support efficacy analyses. Safety analyses were performed in the safety population, defined as all patients who received at least one dose of the study treatment.

The primary and secondary efficacy endpoints were analyzed using repeated measures ANOVA (Wallenstein design), followed by post hoc Dunnett’s *t*-test for multiple comparisons.

For safety measures, adverse events reported in patient diaries were coded by the clinical research organization, using the latest version of the MedDRA dictionary (V27.1) into Preferred Terms (PT) and System Organ Classes (SOC) [64]. The total number of events per patient was provided. Each patient was counted only once within each SOC category and once within each PT category. All prior and concomitant medications were coded according to the Anatomical Therapeutic Chemical (ATC) classification and summarized descriptively.

### 2.9. Ethical Permission

Ethical approval was given by the Ethics Committee (Comitato Etico Milano Area 1) on 10 July 2023, and by amendment from the Comitato Etico Territoriale Lombardia 1 Milano (Approval No: n.2023/ST/081). Written assent/informed consent was obtained from each participant or their legally authorized representative prior to any eligibility assessments. The study adhered to the principles of International Conference on Harmonization Good Clinical Practice (ICH GCP E6 R2) and complied with all applicable laws and regulations.

## 3. Results

### 3.1. Participants

A total of 21 patients (12 males, 9 females; 81% with classical PKU [cPKU] and 19% mild hyperphenylalaninemia [mHPA]) with a mean age of 29.8 ± 8.9 years (range: 17–42 years) were recruited from a single PKU treatment center (Clinica Pediatrica Ospedale San Paolo—ASST Santi Paolo e Carlo; Milan, Italy) and randomly assigned to one of two treatment sequences (AB or BA) (Figure 1, Supplementary Figure S1). Baseline characteristics of participants are summarized in Table 1.

Due to the absence of baseline blood spot samples required for the determination of amino acid levels, five patients were excluded from the efficacy (ITT) analysis, as no evaluable efficacy data were available. Overall, data of the remaining 16 patients (10 males and 6 females; 75% cPKU) with a mean age of 30.3 ± 9.1 years (range: 18–42 years) were included in the ITT analysis.

Four additional patients were excluded from the per-protocol (PP) population due to non-compliance with study procedures. Consequently, 12 patients (8 males and 4 females; 67% cPKU) were included in the PP analysis (31.3 ± 8.6 years; range: 18–42 years).

### 3.2. Primary Outcome: Blood Phe Levels

**Figure 2:**
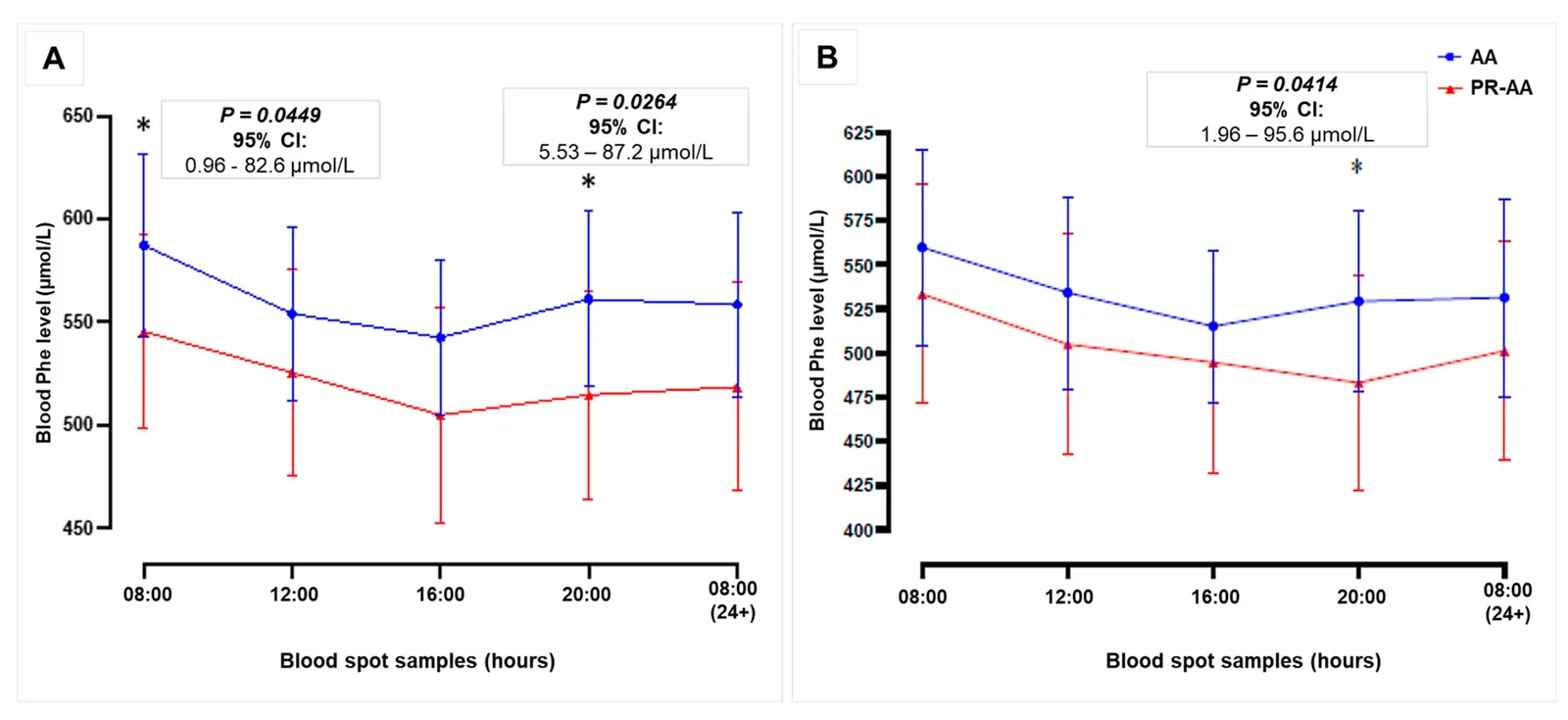
24-h blood Phe profile measured at five time points after treatment with PR-AA (red) and SC (blue); (**A**) efficacy results in intent-to-treat (ITT) population; (**B**) efficacy results in per protocol (PP) population. Abbreviations: SC, standard of care; PR-AA, prolonged-release protein substitute (test product); Phe, phenylalanine; CI: confidence interval.

Mean post-treatment blood Phe levels measured at five time points over a 24 h period are presented in Table 2 and Figure 2. In the ITT population (*n* = 16), treatment with PR-AA over two consecutive days resulted in significantly lower post-treatment blood Phe levels compared with the SC (522 ± 194.4 µmol/L vs. 561 ± 165.2 µmol/L; mean difference: 38.9 µmol/L; 6.9%; p = 0.0009). Results in the PP population (*n* = 12) were consistent with the ITT analysis (504 ± 208.3 µmol/L vs. 534 ± 175.5 µmol/L; mean difference: 30.5 µmol/L; 5.7%; p = 0.0099). No carryover effect was observed for either analysis (p = 0.7702 and p = 0.908).

In both treatments, blood Phe levels were highest in the morning (08:00), declined throughout the day, reached a nadir in the afternoon, then showed a slight increase in the evening and at the end of 24 h. However, the difference between the time profile curves was particularly more pronounced between two treatments at 08:00 (ITT: p = 0.0449; 95% CI: 0.96–82.6 µmol/L) and at 20:00 (ITT: p = 0.0264; 95% CI: 5.53–87.2 µmol/L; PP: p = 0.0414; 95% CI: 1.96–95.6 µmol/L).

### 3.3. Secondary Study Outcomes

#### 3.3.1. Blood Tyr Levels

**Figure 3:**
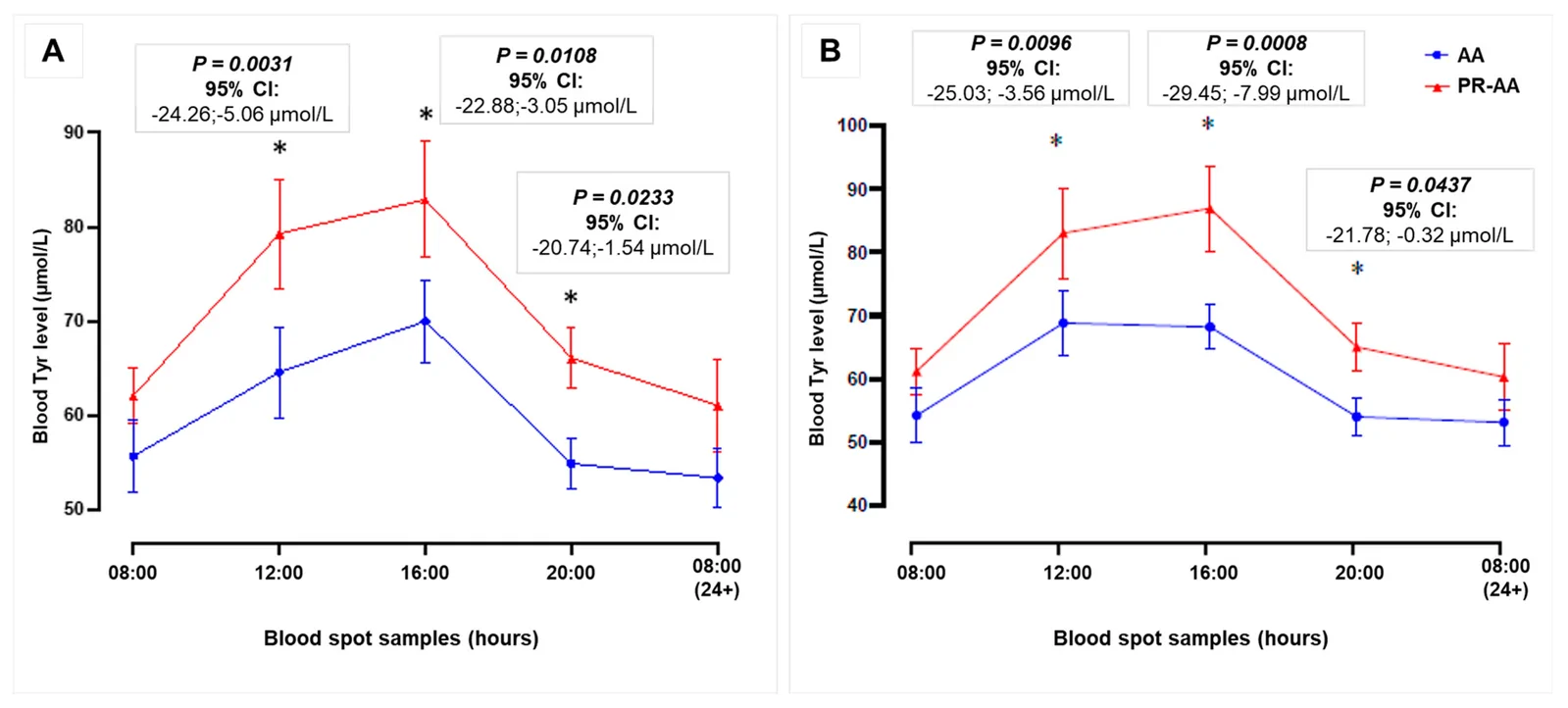
24 h blood Tyr profile measured at five time points after treatment with PR-AA (red) and SC (blue); (**A**) efficacy results in intent-to-treat (ITT) population; (**B**) efficacy results in per protocol (PP) population. Abbreviations: SC, standard of care; PR-AA, prolonged-release protein substitute (test product); Tyr, tyrosine; CI: confidence interval.

Short-term treatment with PR-AA resulted in approximately 18–20% higher blood Tyr levels compared with SC in both the ITT and PP populations (p < 0.05), with no carryover effect detected (p > 0.05) (Table 3). Across the 24 h assessment period, Tyr levels increased from morning to a peak in the afternoon and declined thereafter in both treatment groups (Figure 3). However, PR-AA consistently maintained higher Tyr concentrations across most sampling time points, with significant between-treatment differences observed at 12:00, 16:00, and 20:00. Overall, these findings indicate a sustained increase in blood Tyr levels with PR-AA compared with SC throughout the day.

#### 3.3.2. Phe/Tyr Ratio

**Figure 4:**
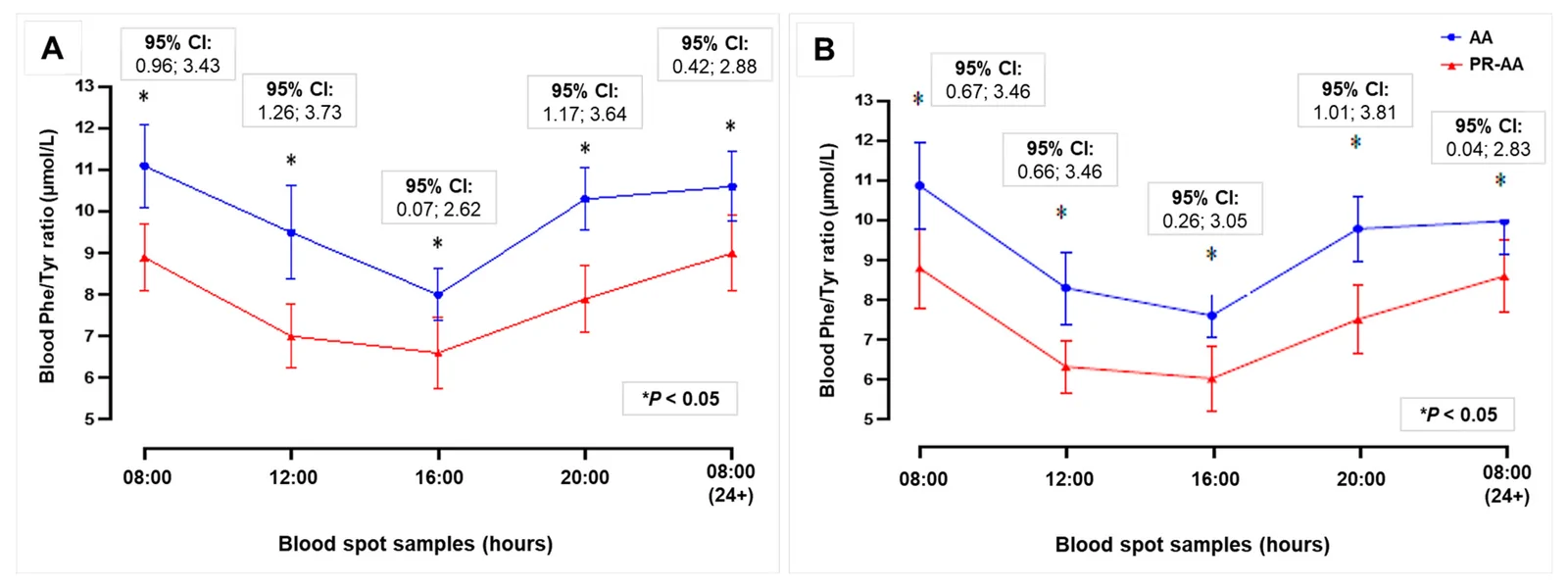
24 h blood Phe/Tyr profile measured at five time points after treatment with PR-AA (red) and SC (blue); (**A**) efficacy results in intent-to-treat (ITT) population; (**B**) efficacy results in per protocol (PP) population. Abbreviations: SC, standard of care; PR-AA, prolonged-release protein substitute (test product); Phe, phenylalanine; Tyr, tyrosine; CI: confidence interval.

The Phe/Tyr ratio was significantly lower with PR-AA compared with SC in both ITT and PP populations (Table 4), with an overall reduction of approximately 20% (p < 0.001). Both treatment groups showed a similar diurnal pattern, with the ratio declining during the day and increasing overnight; however, PR-AA maintained consistently lower values throughout the 24 h assessment period, reflecting the combined effect of lower blood Phe and higher blood Tyr levels (Figure 4).

#### 3.3.3. Blood Branched-Chain Amino Acids (BCAAs)

**Figure 5:**
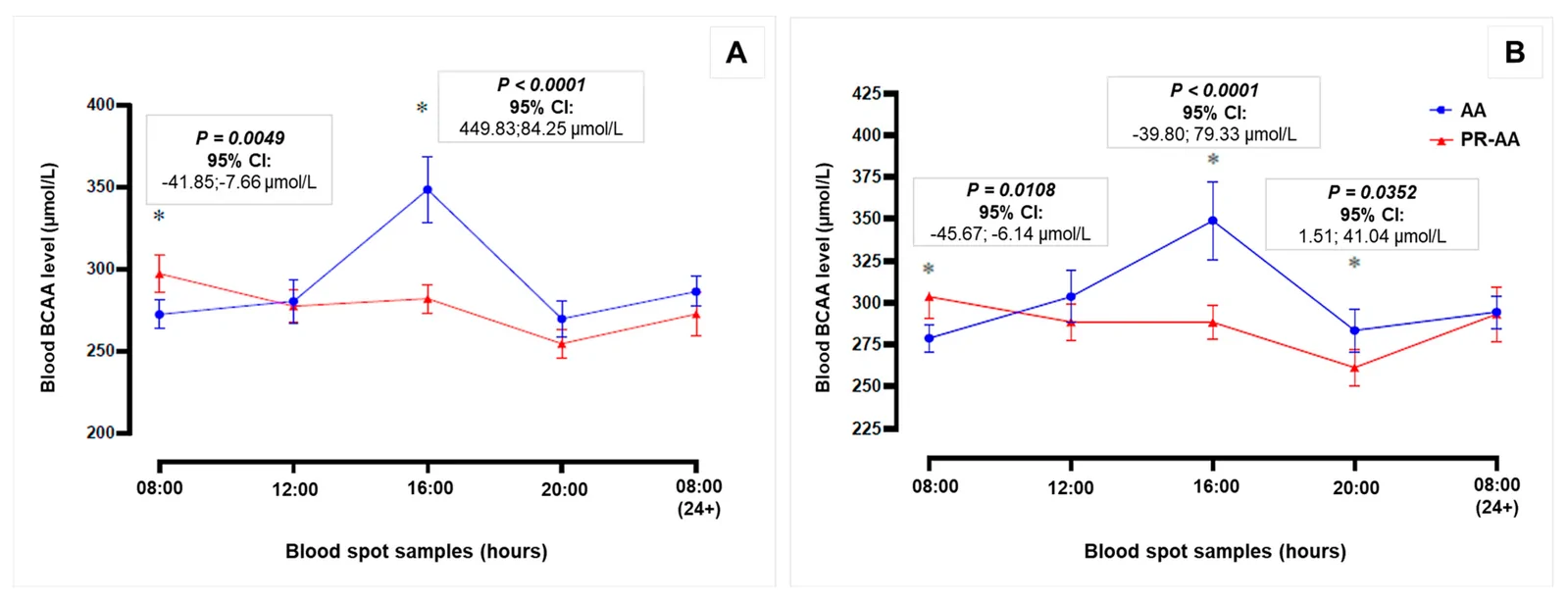
24 h blood BCAA profile measured at five time points after treatment with PR-AA (red) and SC (blue); (**A**) efficacy results in intent-to-treat (ITT) population; (**B**) efficacy results in per protocol (PP) population. Abbreviations: SC, standard of care; PR-AA, prolonged-release protein substitute (test product); BCAA, branched-chain amino acids; CI: confidence interval.

Mean blood BCAA levels were significantly lower (approximately 5%) with PR-AA compared with SC in both ITT and PP populations (Table 5). The most evident difference was observed in the afternoon (at 16:00), when SC treatment showed a pronounced increase in BCAA concentrations, whereas PR-AA displayed a more stable profile without a marked post-prandial surge (Figure 5). No carryover effect was observed (p > 0.05).

### 3.4. Safety

Only one patient experienced an adverse event on the first day of treatment. The reported event was asthenia, classified as non-serious, of mild intensity, and unexpected. The event was assessed as not related to the study treatment. It resolved spontaneously within one day and did not require hospitalization or any specific medical intervention.

## 4. Discussion

In PKU, metabolic control is traditionally defined by maintaining mean blood Phe levels within age-specific therapeutic target ranges to prevent the neurotoxic effects of chronic Phe accumulation [5]. However, unlike the relatively stable Phe profile observed in healthy individuals, blood Phe levels in PKU can fluctuate substantially over 24 h and from day to day [65,66,67,68,69]. Although there is substantial evidence linking long-term Phe variability [26,40,41,44,70] to adverse neurocognitive outcomes, the clinical significance of acute diurnal fluctuations remains unclear. Nevertheless, given the potential cumulative impact of repeated daily Phe oscillations on cerebral exposure over time, therapeutic strategies aimed not only at lowering mean blood Phe but also at improving short-term biochemical parameters of metabolic control may represent a complementary approach to optimizing long-term neurocognitive outcomes in PKU.

As this area remains relatively new, therapeutic strategies specifically aimed at improving short-term biochemical parameters of metabolic control remain limited. The most established approach is the even distribution of protein substitutes throughout the day, typically across at least three to four doses [5]. However, this regimen may compromise adherence, particularly among adolescents and adults, in whom dietary compliance is often suboptimal. Alternative approaches include sapropterin (BH4) therapy and GMP-based protein substitutes, both investigated for their potential effects on Phe stability [24,63,71,72,73]. However, responsiveness to BH4 is largely restricted to milder forms of PKU, while the potential stabilizing effect of GMP may be limited by its residual Phe content [63]. Moreover, a recent kinetic study [74] showed that amino acids derived from cGMP were absorbed more rapidly than casein, indicating that cGMP does not reproduce the slow-release properties of intact proteins. Therefore, although cGMP represents an important peptide-based alternative to free amino acid mixtures, differences in amino acid absorption kinetics remain among currently available protein substitutes.

A novel dietary strategy to improve short-term biochemical parameters of metabolic control in PKU involves protein substitutes designed for prolonged release of amino acids from the gastrointestinal tract [56,57,60]. Previous studies in animal models and healthy volunteers have demonstrated a more sustained absorption profile, improved nitrogen retention, increased blood Tyr availability, and reduced oxidative amino acid losses with such formulations [58,59,60]. Furthermore, a recent randomized controlled crossover study in children with classical PKU (5–16 years) evaluated the effects of PR-AA on diurnal Phe fluctuations compared with conventional protein substitutes [75]. PR-AA resulted in an approximately 18% reduction in early morning fasting blood Phe, achieving a mean concentration of 294 µmol/L, whereas conventional amino acid mixtures were associated with higher morning levels (442 µmol/L), exceeding recommended target ranges. In addition, PR-AA treatment increased Tyr levels and improved Phe/Tyr ratio. However, that study was limited to pediatric patients and assessed only a single evening dose of PR-AA.

The present study is the first randomized, controlled crossover trial in adolescents and adults with PKU evaluating the effects of repeated daily administration of a prolonged-release protein substitute on 24 h blood Phe and Tyr profiles and other short-term biochemical parameters of metabolic control. Three daily doses of PR-AA produced significantly lower mean blood Phe concentrations over 24 h compared with conventional protein substitutes. Although the absolute reduction in mean blood Phe levels was modest (approximately 6–7%), the effect was consistently observed across the 24 h assessment period, particularly during the early morning. Notably, these differences were observed after only two days of treatment, suggesting a rapid metabolic response to the prolonged-release formulation. In addition, PR-AA significantly increased blood Tyr concentrations and reduced the Phe/Tyr ratio at all assessed time points. Circulating BCAA levels were also modestly lower and less variable. Taken together, these findings suggest that a prolonged-release protein substitute may improve short-term biochemical parameters of metabolic control and contribute to more favorable 24 h Phe and Tyr profiles in individuals with PKU, supporting the potential importance of optimizing amino acid delivery kinetics as a therapeutic strategy.

Current dietary management of PKU relies heavily on synthetic amino acid mixtures, which differ substantially from intact protein digestion in terms of absorption rate and metabolic handling. Unlike intact proteins, free amino acid mixtures are rapidly absorbed, resulting in early postprandial plasma peaks followed by relatively rapid declines [49,52]. These kinetic differences may promote transient imbalances between amino acid availability and metabolic demand, potentially contributing to greater short-term fluctuations in blood Phe concentrations. Studies consistently demonstrated an inverse diurnal pattern in PKU, characterized by peak Phe concentrations in the early morning [65,66,68,76,77,78]. This elevation is often attributed to prolonged overnight fasting and increased endogenous protein catabolism. In this study, both treatments showed the expected diurnal pattern, higher Phe levels in the morning and a decline during the day with lower concentrations in the afternoon and evening. Although the resulting mean and median levels remained within the therapeutic target range (120–600 μmol/L), PR-AA consistently maintained lower Phe concentrations at all measured time points over the 24 h period, with statistically significant differences most evident at 08:00 and 20:00. This finding is consistent with the results of the previous study comparing the efficacy of PR-AA and conventional free amino acid-based protein substitutes in reducing morning Phe levels in children with classical PKU [75]. The present data suggest that modifying amino acid delivery kinetics, without altering total prescribed protein equivalent, can be used as a strategy to reduce early morning peaks.

Beyond Phe reduction, PR-AA treatment also resulted in significantly higher and sustained Tyr concentrations throughout the 24 h period. The ability to ensure adequate Tyr availability is clinically relevant, as Tyr represents a direct precursor of neurotransmitters such as dopamine, norepinephrine, and epinephrine, and its deficiency has been linked to impaired neurocognitive performance [1,2].

The combined effect of reduced Phe and increased Tyr contributed to a significant reduction in the Phe/Tyr ratio, a parameter increasingly recognized as more predictive than Phe levels alone for several neuropsychological outcomes. Elevated Phe competitively inhibits cerebral uptake of Tyr via the LAT1 transporter, thereby reducing cerebral catecholamine synthesis [79]. Previous studies have reported associations between higher Phe/Tyr ratios and poorer cognitive and executive functioning in early-treated PKU [46,47]. Therefore, the improvement in Phe/Tyr ratio observed with PR-AA may reflect a metabolic profile potentially more favorable for neurocognitive functioning, although no functional endpoints were assessed in the present study.

An exploratory finding of this study was the modest reduction in circulating BCAA levels with PR-AA, together with a less pronounced afternoon increase compared with conventional protein substitutes. Although the physiological and clinical significance of these findings remains unclear, the results may suggest differences in amino acid handling related to the prolonged-release formulation. Given the exploratory nature of this endpoint, these observations should be interpreted cautiously and require confirmation in larger and longer-term studies.

Overall, the findings of this study suggest that prolonged-release protein substitutes may represent a promising complementary strategy for improving short-term metabolic control in PKU by providing modest but consistently lower blood Phe levels and sustained improvements in blood Tyr levels and the Phe/Tyr ratio across the 24 h assessment period. However, the potential clinical or neurocognitive relevance of these metabolic changes remains to be established. Palatability and product preference were not formally evaluated. Nevertheless, the fiber-based coating of the PR-AA formulation masks the taste and odor of free amino acids. Whether this characteristic translates into improved treatment adherence remains a hypothesis and requires formal evaluation in future studies. Taken together, these findings suggest that a prolonged-release formulation may help improve short-term metabolic control in adolescents and adults without increasing dosing amount or frequency. Given the preliminary nature of these findings and the limited number of participants who completed the study protocol, larger studies with longer follow-up are required to determine whether these favorable changes in 24 h Phe and Tyr profiles translate into meaningful clinical benefits.

Key strengths of this study include the randomized crossover design, which minimizes inter-individual variability, and intensive 24 h sampling to characterize diurnal amino acid dynamics. The absence of carryover effects strengthens internal validity, and consistent findings in both ITT and PP analyses support the robustness of the results.

Several limitations should be acknowledged. The sample size was modest, and only 12 participants completed the study according to the protocol. Although the crossover design increased statistical efficiency by allowing each participant to serve as their own control, the limited number of participants who completed the study warrants cautious interpretation of the findings and highlights the need for confirmation in larger, adequately powered studies. The study population included patients with mHPA, who typically exhibit less pronounced Phe fluctuations than individuals with cPKU. As a result, treatment differences may have been attenuated, and larger effects might be expected in a cohort consisting exclusively of patients with more severe forms. Furthermore, seven participants received cGMP-based protein substitutes as their standard of care. Although this reflects routine clinical practice, free amino acid–based and cGMP-based protein substitutes are not physiologically equivalent comparators. The present study was not designed or powered to compare PR-AA separately with these two categories of protein substitutes. Nevertheless, an exploratory post hoc subgroup analysis stratified according to the type of standard-of-care protein substitute was performed (Supplementary Table S3). In the ITT population, participants receiving cGMP-based protein substitutes as standard of care showed nominally lower blood Phe levels and Phe/Tyr ratios, and higher blood Tyr levels during PR-AA treatment compared with their standard treatment; however, these findings were not reproduced in the PP population. Given the small subgroup sizes and the non-prespecified exploratory nature of these analyses, the findings should be interpreted with caution and regarded as hypothesis-generating only. Accordingly, the present findings should not be interpreted as demonstrating superiority of PR-AA over cGMP-based protein substitutes. Instead, they provide preliminary evidence supporting the potential of prolonged-release amino acid technology under the real-world standard-of-care conditions evaluated in this study.

The short intervention duration (two days per treatment) precludes conclusions regarding long-term metabolic adaptation, adherence, or clinical outcomes. Neurocognitive parameters, nitrogen balance, and body composition were not assessed. Dietary intake outside the protein substitute followed standard care and was not tightly controlled; therefore, real-world variability over longer periods may differ. Finally, although statistically significant, the clinical magnitude of certain changes, particularly in BCAAs, requires further investigation to determine long-term relevance.

Longer-term studies are needed to evaluate whether sustained PR-AA use improves time within target Phe range, reduces intra-individual variability, enhances neurocognitive performance, supports muscle mass preservation, or improves quality of life. Studies incorporating continuous or more frequent Phe monitoring, stable isotope tracer methodologies to assess protein turnover, and comprehensive neuropsychological assessments would provide deeper mechanistic and clinical insight.

## 5. Conclusions

In conclusion, the overall data support the safety and therapeutic potential of PR-AA in the dietary management of PKU. In adolescents and adults, short-term treatment with PR-AA resulted in modest, rapid, and consistent improvements in 24 h metabolic control parameters compared with conventional protein substitutes, including lower blood Phe and higher blood Tyr levels, and a reduced Phe/Tyr ratio.

These findings suggest that optimizing amino acid delivery kinetics may represent a promising approach for achieving more favorable 24 h Phe and Tyr profiles in individuals with PKU and may offer an alternative approach to optimizing amino acid delivery kinetics in PKU. However, given the preliminary nature of these short-term findings, the limited number of participants and the absence of direct clinical or functional endpoints, the broader clinical relevance of these findings remains to be established. Further long-term studies in larger patient populations are required to confirm these findings.

## Figures and Tables

**Table 1:** Baseline characteristics of the participants in Intention-to-Treat Population (ITT) ^a^ and Per Protocol (PP) Population ^b^.

Baseline Characteristics	ITT Population (N = 16)	PP Population (N = 12)	All Participants (N = 21)
**Age at enrollment (years)**			
Mean ± SD	30.3 ± 9.1	31.3 ± 8.6	29.8 ± 8.9
Median [range]	31.5 (18–42)	32 (18–42)	31 (17–42)
**Sex, N (%)**			
Male	10 (62.5)	8 (66.7)	12 (57.1)
Female	6 (37.5)	4 (33.3)	9 (42.9)
**Ethnicity, N (%)**			
Caucasian	16 (100.0)	12 (100.0)	21 (100.0)
**PKU severity, N (%)**			
Classical PKU	12 (75.0)	8 (66.7)	17 (81.0)
Mild HPA ^c^	4 (25.0)	4 (33.3)	4 (19.0)
**Weight (kg)**			
Mean ± SD	70.0 ± 16.4	68.8 ± 15.5	66.9 ± 15.5
Median [range]	72.0 (46.4–104.4)	67.9 (49.0–104.4)	62.3 (46.4–104.4)
**Height (cm)**			
Mean ± SD	166.1 ± 7.7	166.6 ± 15.5	165.8 ± 7.0
Median [range]	166.0 (157–177)	166.0 (157–177)	165.0 (154–177)
**BMI (kg/m^2^)**			
Mean ± SD	25.3 ± 5.1	24.7 ± 5.0	24.2 ± 4.9
Median [range]	24.6 (16.8–34.5)	24.3 (16.8–34.5)	22.5 (16.8–34.5)

^a^ The intention-to-treat (ITT) population included all patients who received at least one dose of the study treatment, irrespective of protocol deviations. ^b^ The per-protocol (PP) population comprised patients with adequate treatment compliance and complete primary efficacy data, without major protocol deviations, and was used to support efficacy analyses. ^c^ The participants classified as mild HPA were categorized according to their neonatal blood Phe levels; however, at study enrolment they required dietary treatment and protein substitute supplementation to maintain blood Phe levels within the recommended therapeutic range. Abbreviations: N, sample size; SD, standard deviation; PKU, phenylketonuria; HPA, hyperphenylalaninemia; BMI, body mass index.

**Table 2:** Blood Phe levels by treatment (PR-AA vs. SC), period, and sampling time point in the ITT and PP populations.

	Treatment	Period	Sample	ITT Population (*n* = 16)			PP Population (*n* = 12)		
				N	Mean (SD)	Median (Range)	N	Mean (SD)	Median (Range)
Aggregated Blood Phe Data	PR-AA			79	522.1 (194.4)	528 (219–953)	60	503.5 (208.3)	483 (219–953)
	SC			79	561.0 (165.2)	576 (221–866)	60	534.0 (175.5)	534 (221–866)
Blood Phe Levels Paired by Sampling Time Point	PR-AA		08:00	16	545.4 (187.5)	560 (280–896)	12	533.4 (214.4)	524 (280–896)
			12:00	16	525.5 (199.5)	540 (253–838)	12	505.0 (216.6)	457 (253–838)
			16:00	15	505.0 (203.2)	528 (219–953)	12	494.7 (218.4)	467 (219–953)
			20:00	16	514.8 (202.4)	515 (221–855)	12	483.2 (210.0)	443 (221–855)
			08:00 (+24)	16	518.7 (202.6)	493 (219–821)	12	501.2 (215.5)	471 (219–821)
	SC		08:00	16	587.2 (177.2)	603 (257–866)	12	559.9 (192.0)	541 (257–866)
			12:00	16	554.1 (167.3)	585 (221–838)	12	534.2 (188.6)	536 (221–838)
			16:00	15	542.6 (147.2)	546 (259–766)	12	515.2 (148.7)	521 (259–766)
			20:00	16	561.1 (170.4)	579 (263–854)	12	529.3 (178.5)	541 (263–854)
			08:00 (+24)	16	558.6 (179.4)	575 (247–834)	12	531.5 (194.1)	527 (247–834)
Blood Phe Levels Stratified by Treatment Period and Sample Collection Time Points	PR-AA	1	08:00	8	553.4 (231.0)	524 (280–896)	7	550.7 (249.4)	484 (280–896)
			12:00	8	523.2 (216.9)	480 (285–838)	7	515.6 (233.1)	384 (285–838)
			16:00	7	505.5 (244.9)	398 (253–953)	7	505.5 (244.9)	398 (253–953)
			20:00	8	509.7 (229.2)	456 (224–855)	7	504.2 (247.0)	384 (224–855)
			08:00 (+24)	8	506.1 (222.7)	483 (219–821)	7	506.2 (240.6)	460 (219–821)
		2	08:00	8	537.4 (147.9)	589 (296–680)	5	509.1 (178.7)	621 (296–661)
			12:00	8	527.9 (195.5)	540 (253–801)	5	490.1 (216.9)	530 (253–801)
			16:00	8	504.7 (176.5)	533 (219–721)	5	479.5 (201.7)	538 (219–721)
			20:00	8	519.9 (187.4)	535 (221–833)	5	453.9 (167.0)	503 (221–631)
			08:00 (+24)	8	531.4 (194.8)	528 (247–799)	5	494.1 (202.0)	481 (247–783)
	SC	1	08:00	8	620.2 (154.6)	669 (297–803)	5	570.8 (172.0)	663 (297–710)
			12:00	8	574.9 (127.6)	590 (294–700)	5	545.8 (153.3)	594 (294–698)
			16:00	8	559.9 (141.6)	577 (259–742)	5	504.6 (145.2)	546 (259–630)
			20:00	8	589.8 (144.4)	609 (297–781)	5	525.7 (135.9)	562 (297–629)
			08:00 (+24)	8	585.1 (158.9)	619 (250–783)	5	532.9 (173.3)	618 (250–685)
		2	08:00	8	554.2 (202.3)	531 (257–866)	7	552.1 (218.4)	493 (257–866)
			12:00	8	533.3 (206.7)	500 (221–838)	7	525.9 (222.0)	455 (221–838)
			16:00	7	522.8 (162.3)	484 (353–766)	7	522.8 (162.3)	484 (353–766)
			20:00	8	532.5 (198.7)	523 (263–854)	7	531.8 (214.6)	508 (263–854)
			08:00 (+24)	8	532.0 (205.1)	517 (247–834)	7	530.5 (221.5)	492 (247–834)

Abbreviations: Phe, phenylalanine; PR-AA, prolonged release protein substitute; SC, standard of care; ITT, intention-to-treat analysis; PP, per protocol analysis; N, sample size; SD, standard deviation.

**Table 3:** Blood Tyr levels by treatment (PR--AA vs. SC), period, and sampling time point in the ITT and PP populations.

	Treatment	Period	Sample	ITT Population (*n* = 16)			PP Population (*n* = 12)		
				N	Mean (SD)	Median (Range)	N	Mean (SD)	Median (Range)
Aggregated Blood Tyr Data	PR-AA			79	70.2 (20.5)	68 (37–149)	60	71.3 (21.7)	69 (37–149)
	SC			79	59.6 (16.1)	58 (32–115)	60	59.6 (15.3)	59 (32–100)
Blood Tyr Levels Paired by Sampling Time Point	PR-AA		08:00	16	62.1 (11.9)	58 (50–88)	12	61.2 (12.6)	56 (50–88)
			12:00	16	79.3 (23.2)	77 (50–149)	12	83.0 (24.8)	77 (56–149)
			16:00	15	82.9 (23.9)	78 (53–128)	12	86.9 (23.5)	81 (53–128)
			20:00	16	66.1 (13.0)	64 (45–86)	12	65.0 (13.0)	64 (45–86)
			08:00 (+24)	16	61.1 (19.6)	55 (37–103)	12	60.3 (18.1)	55 (37–92)
	SC		08:00	16	55.7 (15.0)	51 (36–84)	12	54.2 (15.0)	48 (36–84)
			12:00	16	64.6 (19.2)	69 (32–100)	12	68.8 (18.1)	69 (42–100)
			16:00	15	70.0 (17.0)	64 (53–115)	12	68.2 (12.3)	65 (55–89)
			20:00	16	54.9 (11.0)	59 (36–72)	12	54.0 (10.5)	59 (36–64)
			08:00 (+24)	16	53.4 (12.4)	49 (32–77)	12	53.1 (12.7)	49 (32–77)
Blood Tyr Levels Stratified by Treatment Period and Sample Collection Time Points	PR-AA	1	08:00	8	64.6 (10.4)	65 (50–76)	7	63.0 (10.1)	58 (50–76)
			12:00	8	83.7 (28.9)	75 (58–149)	7	86.8 (29.7)	77 (58–149)
			16:00	7	85.6 (19.0)	85 (65–117)	7	85.6 (19.0)	85 (65–117)
			20:00	8	63.7 (13.2)	60 (48–86)	7	64.9 (13.7)	60 (48–86)
			08:00 (+24)	8	65.1 (18.7)	62 (41–92)	7	66.1 (19.9)	67 (41–92)
		2	08:00	8	59.7 (13.4)	52 (50–88)	5	58.6 (16.4)	52 (50–88)
			12:00	8	74.8 (16.7)	78 (50–100)	5	77.7 (17.6)	78 (56–100)
			16:00	8	80.6 (28.7)	75 (53–128)	5	88.6 (31.1)	78 (53–128)
			20:00	8	68.5 (13.2)	67 (45–83)	5	65.1 (13.6)	65 (45–83)
			08:00 (+24)	8	57.2 (21.0)	49 (37–103)	5	52.1 (12.9)	50 (37–71)
	SC	1	08:00	8	56.6 (15.3)	57 (36–77)	5	52.6 (13.4)	57 (36–70)
			12:00	8	57.0 (17.3)	59 (32–77)	5	59.5 (15.5)	69 (42–74)
			16:00	8	68.3 (20.1)	63 (53–115)	5	62.8 (7.8)	62 (55–74)
			20:00	8	59.0 (11.7)	62 (36–72)	5	56.9 (12.1)	62 (36–64)
			08:00 (+24)	8	52.8 (14.3)	51 (32–72)	5	50.4 (15.0)	46 (32–68)
		2	08:00	8	54.8 (15.8)	48 (42–84)	7	55.3 (17.0)	47 (42–84)
			12:00	8	72.2 (18.8)	69 (48–100)	7	75.4 (17.9)	69 (48–100)
			16:00	7	72.0 (13.9)	75 (56–89)	7	72.0 (13.9)	75 (56–89)
			20:00	8	50.9 (9.3)	50 (40–62)	7	51.9 (9.5)	53 (40–62)
			08:00 (+24)	8	54.1 (11.1)	49 (44–77)	7	55.1 (11.6)	49 (44–77)

Abbreviations: PR-AA, prolonged release protein substitute; SC, standard of care; ITT, intention-to-treat analysis; PP, per protocol analysis; N, sample size; SD, standard deviation.

**Table 4:** Blood Phe/Tyr ratios by treatment (PR-AA vs. SC), period, and sampling time point in the ITT and PP populations.

	Treatment	Period	Sample	ITT Population (*n* = 16)			PP Population (*n* = 12)		
				N	Mean (SD)	Median (Range)	N	Mean (SD)	Median (Range)
Aggregated Phe/Tyr Data	PR-AA			79	7.9 (3.6)	9.7 (2.2–22.0)	60	7.4 (3.1)	7.2 (1.9–15.2)
	SC			79	9.9 (3.4)	7.6 (1.9–17.4)	60	9.3 (3.1)	9.1 (2.2–16.6)
Phe/Tyr Ratios Paired by Sampling Time Point	PR-AA		08:00	16	8.9 (3.2)	7.8 (3.9–15.2)	12	8.8 (3.5)	7.6 (3.9–15.2)
			12:00	16	7.0 (3.1)	6.4 (1.9–15.3)	12	6.3 (2.3)	5.5 (1.9–9.4)
			16:00	15	6.6 (3.3)	4.7 (2.2–13.1)	12	6.0 (2.9)	4.7 (2.2–10.9)
			20:00	16	7.9 (3.2)	7.4 (3.2–14.1)	12	7.5 (3.0)	7.0 (3.2–14.1)
			08:00 (+24)	16	9.0 (3.6)	8.8 (2.4–17.4)	12	8.6 (3.2)	9.6 (2.4–13.2)
	SC		08:00	16	11.1 (4.0)	10.9 (3.1–19.3)	12	10.9 (3.8)	11.2 (3.1–16.6)
			12:00	16	9.5 (4.5)	9.4 (2.2–22.0)	12	8.3 (3.2)	8.5 (2.2–12.5)
			16:00	15	8.0 (2.4)	8.5 (4.8–14.0)	12	7.6 (1.9)	8.4 (4.8–9.8)
			20:00	16	10.3 (3.0)	9.8 (4.9–16.2)	12	9.8 (2.8)	9.6 (4.9–15.4)
			08:00 (+24)	16	10.6 (3.3)	10.5 (4.3–18.5)	12	10.0 (2.9)	10.1 (4.3–14.6)
Phe/Tyr Ratios Stratified by Treatment Period and Sample Collection Time Points	PR-AA	1	08:00	8	8.6 (3.6)	7.7 (3.9–15.2)	7	8.8 (3.8)	7.8 (3.9–15.2)
			12:00	8	6.7 (2.7)	7.2 (1.9–9.4)	7	6.3 (2.7)	5.7 (1.9–9.4)
			16:00	7	6.3 (3.5)	4.7 (2.2–10.9)	7	6.3 (3.5)	4.7 (2.2–10.9)
			20:00	8	8.2 (3.5)	8.6 (3.2–14.1)	7	7.9 (3.7)	7.5 (3.2–14.1)
			08:00 (+24)	8	8.1 (3.2)	8.8 (2.4–12.2)	7	8.0 (3.4)	8.8 (2.4–12.2)
		2	08:00	8	9.2 (3.1)	8.0 (5.8–13.3)	5	8.9 (3.4)	7.2 (5.8–13.3)
			12:00	8	7.3 (3.6)	6.2 (4.5–15.3)	5	6.1 (1.9)	5.3 (4.5–9.0)
			16:00	8	6.8 (3.4)	4.7 (4.2–13.1)	5	5.5 (2.1)	4.6 (4.2–9.3)
			20:00	8	7.6 (3.0)	7.0 (4.9–14.0)	5	6.8 (1.8)	6.9 (4.9–9.7)
			08:00 (+24)	8	9.8 (4.0)	9.2 (5.3–17.4)	5	9.5 (3.0)	10.3 (5.3–13.2)
	SC	1	08:00	8	11.6 (4.3)	10.6 (7.3–19.3)	5	11.1 (3.7)	11.6 (7.3–16.6)
			12:00	8	11.0 (4.9)	9.4 (6.8–22.0)	5	9.3 (2.2)	8.7 (6.8–12.5)
			16:00	8	8.5 (2.8)	8.8 (4.8–14.0)	5	7.9 (1.8)	8.8 (4.8–8.8)
			20:00	8	10.1 (2.5)	9.6 (8.4–16.2)	5	9.2 (0.7)	9.3 (8.4–9.9)
			08:00 (+24)	8	11.5 (3.9)	10.8 (7.7–18.5)	5	10.7 (3.2)	10.1 (7.7–14.6)
		2	08:00	8	10.7 (3.9)	10.9 (3.1–16.4)	7	10.7 (4.2)	10.9 (3.1–16.4)
			12:00	8	8.1 (3.8)	8.3 (2.2–12.2)	7	7.5 (3.7)	6.6 (2.2–12.2)
			16:00	7	7.3 (2.0)	7.6 (5.0–9.8)	7	7.3 (2.0)	7.6 (5.0–9.8)
			20:00	8	10.5 (3.5)	11.3 (4.9–15.4)	7	10.3 (3.7)	10.9 (4.9–15.4)
			08:00 (+24)	8	9.8 (2.7)	10.5 (4.3–12.7)	7	9.5 (2.8)	10.1 (4.3–12.7)

Abbreviations: Phe, phenylalanine; Tyr, tyrosine; PR-AA, prolonged release protein substitute; SC, standard of care; ITT, intention-to-treat analysis; PP, per protocol analysis; N, sample size; SD, standard deviation.

**Table 5:** Blood BCAA levels by treatment (PR-AA vs. SC), period, and sampling time point in the ITT and PP populations.

	Treatment	Sample	ITT Population (*n* = 16)			PP Population (*n* = 12)		
			N	Mean (SD)	Median (Range)	N	Mean (SD)	Median (Range)
Aggregated Blood BCAA Data	PR-AA		16	276.7 (94.0)	263 (144–647)	12	287.0 (98.0)	271.2 (167–647)
	SC		16	290.8 (117.8)	267 (105–847)	12	301.9 (118.0)	267.3 (165–847)
Blood BCAA Levels Paired by Sampling Time Point	PR-AA	08:00	16	297.2 (101.2)	281 (174–591)	12	303.7 (103.5)	281 (199–591)
		12:00	16	277.4 (87.6)	268 (152–532)	12	288.6 (84.3)	276 (198–532)
		16:00	15	282.0 (75.7)	276 (170–463)	12	288.4 (80.5)	279 (170–463)
		20:00	16	254.5 (77.8)	247 (144–485)	12	261.3 (83.6)	234 (167–485)
		08:00 (+24)	16	272.6 (117.2)	241 (170–647)	12	293.2 (128.0)	262 (172–647)
	SC	08:00	16	272.4 (79.5)	271 (117–437)	12	278.8 (63.2)	271 (204–437)
		12:00	16	280.4 (121.0)	259 (154–642)	12	303.7 (120.7)	266 (186–642)
		16:00	15	348.5 (171.9)	279 (200–847)	12	349.0 (181.4)	275 (200–847)
		20:00	16	269.7 (99.8)	253 (105–569)	12	283.5 (101.0)	253 (165–569)
		08:00 (+24)	16	286.4 (80.8)	260 (147–469)	12	294.5 (75.4)	260 (207–469)

Abbreviations: BCAA, branched-chain amino acids; PR-AA, prolonged release protein substitute; SC, standard of care; ITT, intention-to-treat analysis; PP, per protocol analysis; N, sample size; SD, standard deviation.

## Data Availability

The data used and/or analyzed in the current study are available from the corresponding author on reasonable request.

## Supplementary Materials

The following supporting information can be downloaded at: https://www.mdpi.com/article/10.3390/nu18162738/s1, Table S1: Nutrition declaration of study products; Table S2: Characteristics of cGMP-based protein substitutes used as standard of care by participants, including brand, daily protein equivalent intake, and estimated daily phenylalanine (Phe) intake from residual cGMP; Figure S1: CONSORT Flow Diagram of Participant Enrollment, Allocation, Follow-up, and Analysis; Table S3: Exploratory descriptive statistics of blood Phe and Tyr levels stratified by standard-of-care protein substitute (GMP vs. Phe-free AA) and treatment (PR-AA vs. SC) in the ITT and PP populations.

## Abbreviations

The following abbreviations are used in this manuscript:

PKU	Phenylketonuria
Phe	Phenylalanine
Tyr	Tyrosine
PR-AA	Prolonged-release amino acid–based protein substitute
SC	Standard of care
BCAAs	Branched-chain amino acids
PAH	Phenylalanine hydroxylase
LNAAs	Large neutral amino acids
cGMP	Casein glycomacropeptide
AA	L-amino-acid–based protein substitute
SD	Standard deviation
CI	Confidence interval
ITT	Intention-to-treat
PP	Per protocol
PT	Preferred Terms
SOC	System Organ Classes
ATC	Anatomical Therapeutic Chemical
ICH GCP	International Conference on Harmonization Good Clinical Practice
cPKU	Classical PKU
mHPA	Mild hyperphenylalaninemia
BMI	Body mass index
BH4	Sapropterin (tetrahydrobiopterin)
