# Supplementary material for: Effects of a Prolonged-Release Protein Substitute on 24-h Phenylalanine and Tyrosine Profiles in Phenylketonuria: A Randomized Crossover Study
Source: Nutrients. 2026 Aug 21;18(16):2738. doi: 10.3390/nu18162738 (PMC13516870; doi:10.3390/nu18162738)
Supplement: Supplementary file 1 [file nutrients-18-02738-s001.zip › nutrients-4426433-supplementary.pdf]

Supplementary Table S1. Nutrition declaration of study products.

	PKU GOLIKE PLUS 16+		PKU GOLUKE KRUNCH	
**Components, units**	**Per 100 g**	**Per sachet of 31.5 g**	**Per 100 g**	**Per tablet of 1.65 g**
**Energy and macronutrient**				
Energy, kcal (kJ)	283 (1200)	90 (380)	341 (1427)	169 (707)
Fat, grams	0.0	0.0	3.0	1.5
of which saturates, g	0.0	0.0	3.0	1.5
Carbohydrate, g	4.0	1.3	14.5	7.2
of which sugars, g	0.0	0.0	12.7	6.3
Fiber, g	7.2	2.3	6.7	0.3
Protein equivalent[1], g	63.2	20.0	60.6	30
Salt, g	0.06	0.02	0.06	0.03
**Amino acids**				
L-serine, g	2.6	0.8	2.5	1.2
L-threonine, g	3.8	1.2	3.6	1.8
L-leucine, g	8.8	2.8	8.4	4.1
Glycine, g	3.8	1.2	3.6	1.8
L-alanine, g	2.3	0.7	2.2	1.1
L-arginine, g	3.0	1.0	2.9	1.4
L-cystine, g	1.5	0.5	1.5	0.7
L-glutamine, g	15.2	4.8	14.6	7.2
L-histidine, g	2.1	0.7	2.0	1.0
L-aspartic acid, g	4.6	1.4	4.4	2.2
L-proline, g	4.6	1.4	4.4	2.2
L-isoleucine, g	4.2	1.3	4.0	2.0
L-lysine, g	5.3	1.7	5.1	2.5
L-tryptophan, g	1.5	0.5	1.5	0.7
L-valine, g	3.8	1.2	3.6	1.8
L-methionine, g	1.1	0.3	1.0	0.5
L-tyrosine, g	7.6	2.4	7.3	3.6
L-phenylalanine, mg	-	-	-	-
**Vitamins**				
Vitamin A (RE), μg	1000	315		
Vitamin D, μg	17	5.4		
Vitamin E, mg	17	5.4		
Vitamin K, μg	100	32		
Vitamin C, mg	150	47.2		
Thiamin, mg	1.7	0.5		
Riboflavin, mg	2.0	0.6		
Niacin, mg	22	6.9		
Vitamin B6, mg	2.4	0.8		

[1] 1g of protein equivalent = 1.2 g of AAs.
The protein content is provided by the amino-acids.

Supplementary Table S1. Cont'.

Component, units	PKU GOLIKE PLUS 16+		PKU GOLUKE KRUNCH	
	Per 100 g	Per sachet of 31.5 g	Per 100 g	Per tablet of 1.65 g
Folic acid, µg	417	131		
Vitamin B12, µg	4.0	1.4		
Biotin, µg	71	23		
Pantothenic acid, mg	6.3	2.0		
**Minerals**				
Potassium, mg	1111	350		
Calcium, mg	1200	378		
Magnesium, mg	350	110		
Phosphorus, mg	950	299		
Chloride, mg	0.69	0.22		
Sodium, mg	25.1	7.9		
Iron, mg	21.0	6.6		
Zinc, mg	14.0	4.5		
Copper, mg	1.4	0.45		
Manganese, mg	2.8	0.88		
Selenium, µg	75	24		
Chromium, µg	50	16		
Molybdenum, µg	60	19		
Iodine, µg	200	63		
**Others**				
Carnitine, g	0.08	0.03	0.08	0.04
Taurine, g	0.21	0.07	0.21	0.10
Choline, g	0.29	0.92		
Inositol, g	0.15	0.47		

Supplementary Table S2. Characteristics of cGMP-based protein substitutes used as standard of care by participants, including brand, daily protein equivalent intake, and estimated daily phenylalanine (Phe) intake from residual cGMP.

Patient Number	Type of protein substitute (standard-of-care)	Brand	Daily Quantity of protein substitute	Daily Quantity of PE intake from protein substitute (g/day)	Phe daily content of cGMP supplementation (mg/day)
01-002 ITT	cGMP	Glytactin RTD Lite+BUILD10	750 ml + 15.9 g	54.9	45.0 + 15.9 = 60.9
01-003	cGMP	Glytactin RTD Lite	750 ml	45.0	45.0
01-009	cGMP	Glytactin RTD 15	750 ml	45.0	78.0
01-010 ITT	cGMP	Glytactin RTD 15	750 ml	45.0	78.0
01-012	cGMP	Glytactin RTD Lite	750 ml	45.0	45.0
01-016	cGMP	Glytactin RTD 15	501 ml	30.0	52.1
01-018	cGMP	Enjoy 10 metaX	111 g	60.0	102.0

Abbreviations: ITT, intention-to-treat population; cGMP, casein glycomacropeptide; PE, protein equivalent; Phe, phenylalanine.

Supplementary Figure S1. CONSORT Flow Diagram of Participant Enrollment, Allocation, Follow-up, and Analysis

CONSORT

TRANSPARENT REPORTING of TRIALS

CONSORT 2010 Flow Diagram

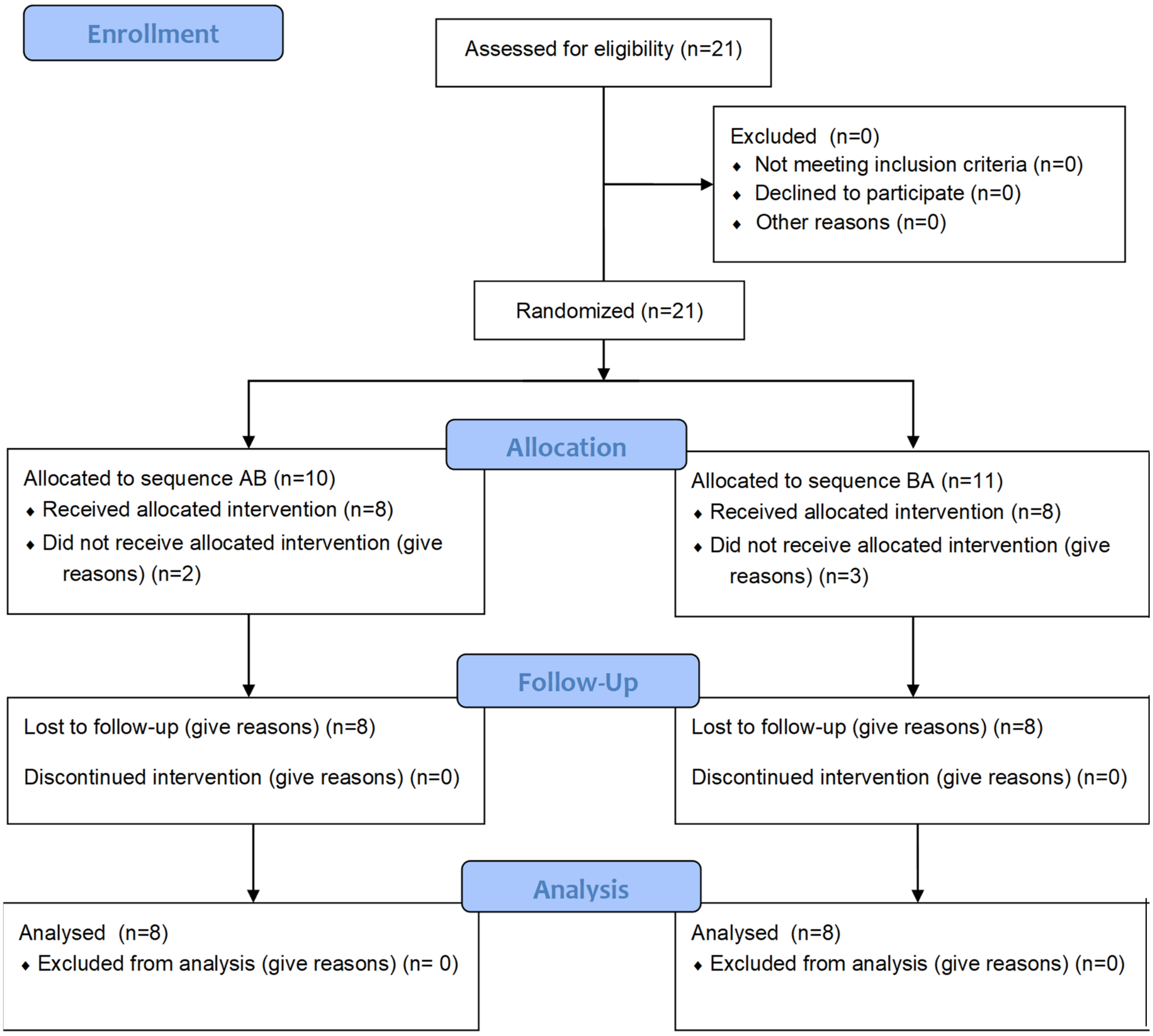

Supplementary Table S3. Exploratory descriptive statistics of blood Phe and Tyr levels stratified by standard-of-care protein substitute (GMP vs. Phe-free AA) and treatment (PR-AA vs. SC) in the ITT and PP populations.

Blood parameter	Standard of care	Treatment	ITT population					PP population[b]		
			N of samples	Mean (SD)	Median [range]	95% CI	P^a	N of samples	Mean (SD)	Median [range]
Blood Phe level	Phe-free AA	SC	25	558.3 (182.8)	597 [221 – 866]	34.78; 116.21	0.0011	20	547 (203.1)	561 [221 – 866]
		PR-AA	25	538.3 (213.6)	550 [219 – 953]			20	529 (238.7)	483 [219 – 953]
	cGMP	SC	29	534.7 (132.2)	562 [250 – 713]			20	503 (144.4)	543 [250 – 710]
		PR-AA	29	444.7 (127.0)	503 [219 – 635]			20	426 (142.6)	444 [219 – 635]
Blood Tyr level	Phe-free AA	SC	25	69.2 (15.7)	74.0 [42.2 – 100.1]	−26.66; 2.16	0.0997	20	69.5 (17.1)	74.1 [42.2 – 100.1]
		PR-AA	25	80.4 (25.2)	77.7 [36.6 – 148.7]			20	81.4 (27.0)	75.7 [36.6 – 148.7]
	cGMP	SC	25	55.0 (16.7)	50.0 [32.2 – 115.0]			20	51.1 (12.1)	48.3 [32.2 – 69.7]
		PR-AA	25	66.2 (17.0)	63.1 [44.9 – 112.7]			20	65.0 (18.0)	59.4 [44.9 – 112.7]
Blood Phe/Tyr ratio	Phe-free AA	SC	25	8.5 (3.1)	8.9 [2.2 – 14.6]	1.59; 4.79	0.0008	20	8.3 (3.4)	9.2 [2.2 – 14.6]
		PR-AA	25	7.4 (3.7)	7.4 [1.9 – 14.1]			20	7.3 (4.1)	6.5 [1.9 – 14.1]
	cGMP	SC	29	10.1 (2.8)	9.9 [4.8 – 16.6]			20	10.1 (3.1)	9.2 [4.8 – 16.6]
		PR-AA	29	6.9 (2.0)	7.2 [4.2 – 10.5]			20	6.7 (2.0)	6.1 [4.2 – 10.5]

[a] Adjusted P value for the treatment effect stratified by standard-of-care (SOC) protein substitute.

[b] No statistically significant treatment differences according to standard-of-care (SOC) protein substitute were observed in the PP population.

Abbreviations: Phe, phenylalanine; Phe-free AA, Phe-free amino acid-based protein substitute; cGMP, casein glycomacropeptide-based protein substitute; PR-AA, prolonged release protein substitute; SC, standard of care; ITT, intention-to-treat analysis; PP, per protocol analysis; N, number; SD, standard deviation.
